# Diagnostic and prognostic implications of 2018 guideline for the diagnosis of idiopathic pulmonary fibrosis in clinical practice

**DOI:** 10.1038/s41598-021-95728-7

**Published:** 2021-08-13

**Authors:** Jooae Choe, Byoung Soo Kwon, Kyung-Hyun Do, Hee Sang Hwang, Jin Woo Song, Eun Jin Chae

**Affiliations:** 1 Department of Radiology and Research Institute of Radiology, University of Ulsan College of Medicine, Asan Medical Center, 88 Olympic-ro 43 Gil, Songpa-gu, Seoul, South Korea; 2 Division of Pulmonary and Critical Care Medicine, Department of Internal Medicine, Seoul National University Bundang Hospital, Seongnam-Si, Gyeonggi-Do South Korea; 3 Department of Pathology, University of Ulsan College of Medicine, Asan Medical Center, 88 Olympic-ro 43 Gil, Songpa-gu, Seoul, South Korea; 4 Department of Pulmonology and Critical Care Medicine, University of Ulsan College of Medicine, Asan Medical Center, 88 Olympic-ro 43 Gil, Songpa-gu, Seoul, South Korea

**Keywords:** Medical research, Respiratory tract diseases

## Abstract

The purpose of this study was to evaluate the implications of the 2018 updated guideline for the diagnosis of idiopathic pulmonary fibrosis (IPF) in clinical practice compared to 2011 guideline. This study involved 535 patients including 339 IPF and 196 non-IPF, and we retrospectively evaluated CT classifications of usual interstitial pneumonia (UIP) by two guidelines. Interobserver agreement of 2018 criteria showed moderate reliability (κ = 0.53) comparable to 2011 (κ = 0.56) but interobserver agreement for probable UIP was fair (κ = 0.40). CT pattern of indeterminate for UIP was associated with better prognosis compared with the other groups (adjusted hazard ratio [HR] = 0.36, *p* < 0.001). Compared to possible UIP, probable UIP demonstrated a lower positive predictive value (PPV, 62.9% vs 65.8%). In analysis of patients with CT patterns of non-definite UIP, diagnosing IPF when CT pattern showed probable UIP with lymphocyte count ≤ 15% in BAL fluid, and either male sex or age ≥ 60 years showed a high specificity of 90.6% and a PPV of 80.8% in the validation cohort. The 2018 criteria provide better prognostic stratification than the 2011 in patients with possible UIP. BAL fluid analysis can improve the diagnostic certainty for IPF diagnosis in patients with probable UIP CT pattern.

## Introduction

Idiopathic pulmonary fibrosis (IPF), the most common form of idiopathic interstitial pneumonia (IIP), is a chronic progressive lung disease of unknown cause with significantly worse prognosis than other forms of IIP^1, 2^. Medical treatments for this deadly disease are dependent on the accurate diagnosis of IPF^3, 4^.

Diagnostic guidelines for IPF were updated in 2018 by the American Thoracic Society (ATS), the European Respiratory Society (ERS), the Japanese Respiratory Society (JRS), and the Latin American Thoracic Society (ALAT)^5^. High-resolution computed tomography (HRCT) patterns for usual interstitial pneumonia (UIP) were further refined to patterns for UIP, probable UIP, indeterminate for UIP and alternative diagnoses. In contrast to the recommendation of Fleischner society which were made against performing surgical lung biopsy in patients with newly detected IIP who has a CT pattern of probable UIP^6^, updated ATS/ERS/JRS/ALAT guideline conditionally recommends surgical lung biopsy for the patient who has a CT pattern of probable UIP, that deciding whether or not to undergo biopsy in patients depends on clinical likelihood of IPF^5, 7^. Moreover, performing bronchoalveolar lavage (BAL) is also conditionally recommended in group of patients with CT pattern of probable UIP, indeterminate for UIP and alternative diagnosis prior to surgical lung biopsy to distinguish IPF from the other alternative diagnosis including eosinophilic pneumonia, sarcoidosis, and hypersensitivity pneumonitis (HP). These recommendations, however, are conditional, and it is currently unclear how changes in the guidelines for IPF will affect their diagnostic performance in real clinical practice.

The purpose of the current study was validate the latest 2018 diagnostic guideline of IPF in the cohort of fibrosing interstitial lung disease (ILD) and evaluate the diagnostic and prognostic implications compared to previous 2011 guideline in clinical practice. The interobserver agreement, diagnostic performance and survival outcomes were compared. Furthermore, the added diagnostic value of cellular analysis of BAL fluid was assessed in patients with CT patterns of probable UIP, indeterminate for UIP and alternative diagnoses following the guidline.

## Results

**Figure 1:**
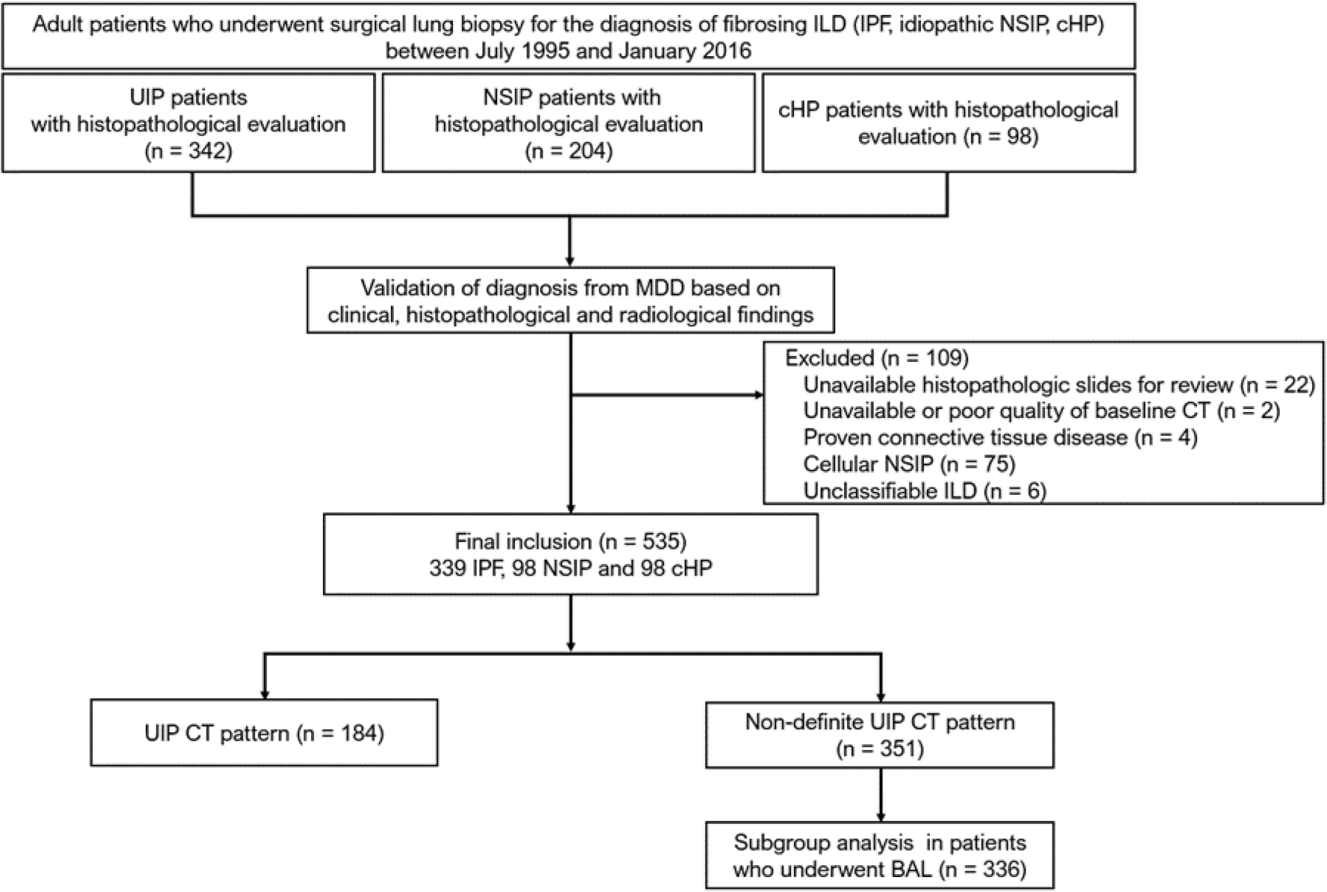
Flow chart of patient inclusion. *ILD* interstitial lung disease; *IPF* idiopathic pulmonary fibrosis; *UIP* usual interstitial pneumonia; *NSIP* nonspecific interstitial pneumonia; *cHP* chronic hypersensitivity pneumonitis; *MDD* multidisciplinary discussion; *BAL* bronchoalveolar lavage.

The study cohort included 535 patients (mean age 60.8 ± 9.0 years, 348 men) with fibrosing ILD (Fig. 1). The clinical characteristics of these patients are summarized in Table 1.

**Table 1:** Demographic and clinical characteristics of patients assorted by diagnostic categories.

Characteristics	IPF (n = 339)	iNSIP (n = 98)	cHP (n = 98)	*p* value^1^	*p* value^2^
Age, years	62.6 ± 0.4	56.4 ± 1.0	58.7 ± 1.1	< 0.001	0.008
Male sex	259 (76.4)	51 (52.0)	38 (38.8)	< 0.001	< 0.001
Ever smoker	233 (68.7)	50 (51.0)	35 (35.7)	0.002	< 0.001
Smoking pack-year, years	35.1 ± 1.4	28.3 ± 2.8	28.6 ± 3.2	0.025	0.086
6 min walking distance, m	466.2 ± 6.6	469.4 ± 14.6	473.4 ± 14.4	0.853	0.721
DL_CO_% predicted	59.9 ± 1.3	55.8 ± 2.8	61.0 ± 3.0	0.041	0.374
FEV_1_% predicted	79.6 ± 1.3	73.9 ± 2.2	81.7 ± 2.3	0.094	0.052
FVC% predicted	72.9 ± 1.3	71.4 ± 2.3	76.5 ± 2.1	0.074	0.230
**Bronchoalveolar lavage**^**†**^					
Lymphocytes (%)	13.2 ± 12.3	21.2 ± 19.5	26.2 ± 19.7	< 0.001	< 0.001
Neutrophils (%)	10.6 ± 17.9	10.2 ± 15.7	5.8 ± 9.1	0.885	0.018
Eosinophils (%)	4.0 ± 5.6	5.2 ± 7.6	3.3 ± 4.6	0.158	0.317

Data are presented as mean ± standard devation or number (%).

UIP, usual interstitial pneumonia; iNSIP, idiopathic nonspecific interstitial pneumonia; cHP, chronic hypersensitivity pneumonitis; DL_CO_, diffusing capacity of the lung for carbon monoxide; FEV_1_, forced expiratory volume in 1 s; FVC, forced vital capacity.

^1^UIP vs iNSIP; ^2^ UIP vs cHP.

^†^Analysed in 220 patients with UIP, 65 patients with iNSIP and 86 patients with cHP.

### Interobserver agreement

Interobserver agreement of 2018 criteria showed moderate reliability (κ = 0.53; range: 0.54‒0.67) comparable to 2011 criteria (κ = 0.56; range: 0.56‒0.70). Interobserver agreement was substantial for UIP (κ = 0.66; range: 0.59‒0.76), fair for probable UIP (κ = 0.40; range: 0.53‒0.60) and moderate for indeterminate for UIP (κ = 0.49; range: 0.39‒0.57) and alternative diagnosis (κ = 0.54; range: 0.47‒0.63). Interobserver agreement across the 2018 diagnostic categories was lower in patients with a more than moderate degree of emphysema than in patients with no or a mild degree of emphysema (Fleiss κ = 0.37 vs 0.55).

### Differences in patient classification and diagnostic performances of the 2011 and 2018 diagsnotic criteria

Assessments of CT patterns and changes in classification in the 535 patients according to the 2011 and 2018 criteria are demonstrated in Table 2. Of the 219 patients with possible UIP according to 2011 criteria, 175 (80.0%) were reclassified as having a probable UIP, 42 (19.2%) as an indeterminate for UIP and two (0.9%) as an alternative diagnosis. The proportion of patients diagnosed with IPF was significantly higher in patients with UIP CT pattern than in those with probable UIP CT pattern (89.7% vs 62.9%; *p* < 0.001), but did not differ significantly in patients with probable UIP compared with patients with indeterminate for UIP (62.9% vs 78.6%; *p* = 0.05; Supplementary table 1).

**Table 2:** Difference in categorization of fibrosing interstitial lung disease based on 2018 and 2011 diagnostic criteria.

CT pattern		2018 criteria				
		UIP	Probable UIP	Indeterminate for UIP	Alternative diagnosis	Total
2011 criteria	UIP	184	0	0	0	184
	Possible UIP	0	175	42	2^†^	219
	Inconsistent with UIP	0	0	0	132	132
	Total	184	175	42	134	535

*CT* computed tomography; *UIP* usual interstitial pneumonia.

^†^Two patients classified as having possible UIP on 2011 criteria showed ancillary findings, including pleural effusion and thickening or dilated esophagus and classified as having a CT pattern of alternative diagnosis on 2018 criteria.

Assessment of its diagnostic performance showed that the CT pattern of UIP had a high specificity (90.3%; 95% confidence interval [CI]: 85.3–94.1%) and positive predictive value (PPV, 89.7%; 95% CI: 84.8–93.1%) but a low sensitivity (48.7%; 95% CI: 43.2–54.1%) for IPF diagnosis (Table 3). Compared with 2011 criteria for possible UIP, the CT pattern of probable UIP had a lower sensitivity (63.2% vs 82.8%), PPV (62.9% vs 65.8%) and negative predictive value (NPV, 63.7% vs 77.3%) but a higher specificity (63.3% vs 57.6%). IPF diagnosis based on CT patterns of UIP and probable UIP showed a lower specificity (57.1%; 95% CI: 49.9–64.2%) and PPV (76.6%; 95% CI: 73.4–79.5%) but a higher sensitivity (81.1%; 95% CI: 76.5–85.1%) than diagnosis based on UIP alone.

**Table 3:** Diagnostic performance according to the CT pattern for the diagnosis of idiopathic pulmonary fibrosis.

Criteria	Sens	Spec	PPV	NPV	Accuarcy
**2011 criteria**					
UIP	48.7 (43.2–54.1)	90.3 (85.3–94.1)	89.7 (84.8–93.1)	50.4 (47.6–53.3)	63.9 (59.7–68.0)
Possible UIP^†^	82.8 (76.3–88.1)	57.6 (50.0–65.0)	65.8 (61.5–69.8)	77.3 (70.6–82.8)	70.1 (65.0–74.8)
UIP and possible UIP	91.2 (87.6–94.0)	52.0 (44.8–59.2)	76.7 (73.9–79.2)	77.3 (70.2–83.1)	76.8 (73.0–80.3)
**2018 criteria**					
UIP	48.7 (43.2–54.1)	90.3 (85.3–94.1)	89.7 (84.8–93.1)	50.4 (47.6–53.3)	63.9 (59.7–68.0)
Probable UIP^†^	63.2 (55.7–70.4)	63.3 (55.7–70.4)	62.9 (57.5–68.0)	63.7 (58.3–68.7)	63.2 (58.0–68.3)
UIP and probable UIP	81.1 (76.5–85.1)	57.1 (49.9–64.2)	76.6 (73.4–79.5)	63.6 (57.6–69.2)	72.3 (68.3–76.1)

^**†**^After excluding patients with CT pattern of UIP.

*PPV* positive predictive value; *NPV* negative predictive value; *AUC* area under the receiver-operating characteristic curve; *UIP* usual interstitial pneumonia.

### Characteristics of patients categorized as interminate for UIP

In the 42 patients with a CT pattern of indeterminate for UIP, 28.6% (12/42) showed CT findings of subtle reticulation with or without mild ground-glass opacities as an early ILD. The other 71.4% (30/42) were truly indeterminate for UIP, the CT patterns and/or distribution of lung fibrosis that did not suggest any specific etiology and among such patients, 10 patients (23.8%, 10/42) showed fibrosis mixed with emphysema.

Among patients with IPF, those indeterminate for UIP showed a longer smoking history than those with other CT patterns (43.8 ± 22.6 vs 34.1 ± 19.2 pack-years; *p* = 0.022). In addition, the percentage with more than a moderate degree of emphysema was significantly higher in patients indeterminate for UIP (27.3%) than in those with UIP (8.5%), probable UIP (6.4%) and alternative diagnoses (6.5%) (*p* = 0.003). Baseline forced vital capacity (FVC, 82.5% ± 19.7% vs 70.1% ± 18.3%; *p* = 0.001), diffusing capacity of the lung for carbon monoxide (DL_CO_, 69.0% ± 23.9% vs 57.3% ± 17.8%; *p* = 0.002) and forced expiratory volume in 1 s (FEV_1_, 86.0% ± 20.7% vs 77.9% ± 18.8%; *p* = 0.029) were also significantly higher in patients with CT pattern of indeterminate for UIP than in those with other CT patterns, suggesting better pulmonary function.

### Survival analysis

**Figure 2:**
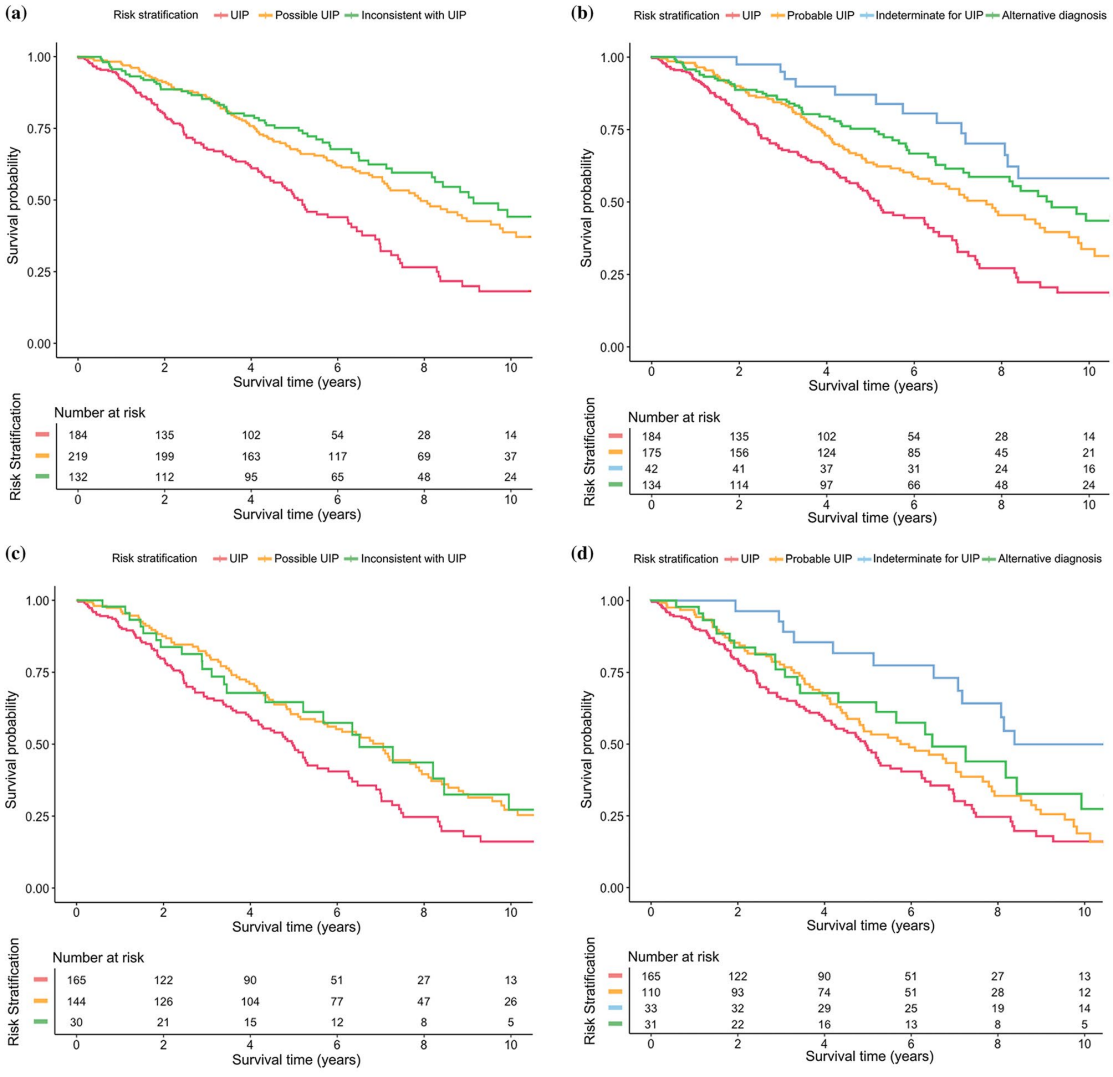
Adjusted survival curves of overall survival stratified according to the CT patterns of 2011 and 2018 guidelines for diagnosis of idiopathic pulmonary fibrosis. **(a**,**b**) Patients with fibrosing interstitial lung disease (total cohort) and (**c**,**d**) patients with idiopathic pulmonary fibrosis as a function of CT pattern according to the 2011 and 2018 diagnostic criteria, respectively. Survival curves were adjusted by age, sex, baseline forced vital capacity, diffusing capacity of the lung for carbon monoxide and use of anti-fibrotics.

Significant prognostic differences were observed when patients were grouped by the HRCT criteria of both the 2011 and 2018 guidelines (log-rank test, *p* < 0.001; Supplementary Fig. 1). Based on 2018 criteria, survival was significantly longer in patients with CT pattern of indeterminate for UIP than in those with the other CT patterns (median survival of patients with CT pattern of indeterminate for UIP, 11.1 years [95% CI: 7.5–14.7] in IPF cohort; 11.5 years [95% CI: 10.5–12.4] in total cohort). After adjusting covariates, patients with CT pattern of indeterminate for UIP and probable UIP showed significantly better prognosis compared with UIP pattern (adjusted hazard ratio [HR] = 0.37 [95% CI: 0.22–0.61], *p* < 0.001 for CT pattern of indeterminate for UIP; adjusted HR = 0.61 [95% CI: 0.46–0.81], *p* = 001 for CT pattern of probable UIP; Table 4) in total patients, regardless of diagnosis (i.e. IPF or non-IPF). In IPF cohort, CT pattern of indeterminate for UIP was associated with better survival compared with UIP pattern (adjusted HR = 0.42 [95% CI: 0.25–0.71], *p* = 0.001) but CT pattern of probable UIP was not significantly associated with survival (adjusted HR = 0.79 [95% CI: 0.58–1.08], *p* = 0.142). Among the patients with CT pattern of probable UIP, survival difference was observed according to diagnosis. Idiopathic nonspecific interstitial pneumonia (iNSIP) showed better prognosis compared to IPF but chronic HP (cHP) showed comparable survival outcome compared to IPF with same CT pattern (adjusted HR = 0.23 [0.12–0.44], *p* < 0.001 for iNSIP; adjusted HR = 0.84 [0.50–2.38], *p* = 0.837 for cHP). Adjusted survival curves according to HRCT crteria of 2011 and 2018 guidelines are shown in Fig. 2.

**Table 4:** Cox proportional hazard analysis for survival time.

Variables	Adjusted hazard ratio (95% CI)	*p* value
**Total patients**		
**2011 criteria** (reference: UIP pattern)		
Possible UIP	0.55 (0.42–0.72)	< 0.001
Inconsistent with UIP	0.45 (0.33–0.62)	< 0.001
**2018 criteria** (reference: UIP pattern)		
Probable UIP	0.61 (0.46–0.81)	0.001
Indeterminate for UIP	0.37 (0.22–0.61)	< 0.001
Alternative diagnosis	0.46 (0.33–0.63)	< 0.001
**IPF only**		
**2011 criteria** (reference: UIP pattern)		
Possible UIP	0.68 (0.50–0.91)	0.009
Inconsistent with UIP	0.66 (0.42–1.03)	0.069
**2018 criteria** (reference: UIP pattern)		
Probable UIP	0.79 (0.58–1.08)	0.142
Indeterminate for UIP	0.42 (0.25–0.71)	0.001
Alternative diagnosis	0.65 (0.41–1.02)	0.062

Hazard ratios were adjusted by age, sex, baseline (% predicted) forced vital capacity (FVC), diffusing capacity of the lung for carbon monoxide (DL_CO_) and use of anti-fibrotics. *CI* confidence interval; *UIP* usual interstitial pneumonia; *IPF* idiopathic pulmonary fibrosis; *iNSIP* idiopathic nonspecific interstitial pneumonia; *cHP* chronic hypersensitivity pneumonitis.

### Added value of BAL fluid analysis in the diagnosis of IPF

To evaluate the added value of BAL fluid analysis in the diagnosis of IPF, the 336 patients with CT patterns of non-definite UIP (probable UIP, indeterminate for UIP, alternative diagnosis) who underwent BAL fluid analysis were randomly divided into development (n = 142) and validation (n = 94) cohorts. Their clinical characteristics is presented on Supplementary table 2.

In the development cohort, median lymphocyte counts in BAL fluid differed significantly between IPF and non-IPF groups (11 [IQR: 3–22] vs 19 [IQR: 10–55]; *p* < 0.001) (Supplementary Fig. 2), but median neutrophil counts were similar (4 [IQR: 1–7] vs 3 [IQR: 1–7]; *p* = 0.815). The optimal cut-off value for lymphocyte counts in BAL fluid for the diagnosis of IPF was 15%. The ability of clinical variables, including age, sex and lymphocyte count in BAL fluid, to predict IPF in the development cohort was tested relative to CT pattern. Older age (≥ 60 years), male sex, low lymphocyte count (≤ 15%) in BAL fluid, and probable UIP pattern were all significant predictors of IPF in univariable and multivariable analysis (Supplementary table 3). Subgroup analysis among the patients with probable UIP CT pattern, showed that male sex and low lymphocyte count (≤ 15%) were significant predictors of IPF in multivariable analysis (Supplementary table 4).

Among the patients with CT patterns of probable UIP who underwent BAL fluid analysis, 73.0% (54/74) in the development cohort and 71.1% (32/45) in the validation cohort were correctly classified as either IPF or non-IPF based on the cut-off value of lymphocyte count of 15% in BAL fluid. Among the patients with CT patterns of probable UIP, 66.2% (49/74) in the development cohort and 46.7% (21/45) were correctly classified as either IPF or non-IPF based on male with age ≥ 60.

**Figure 3:**
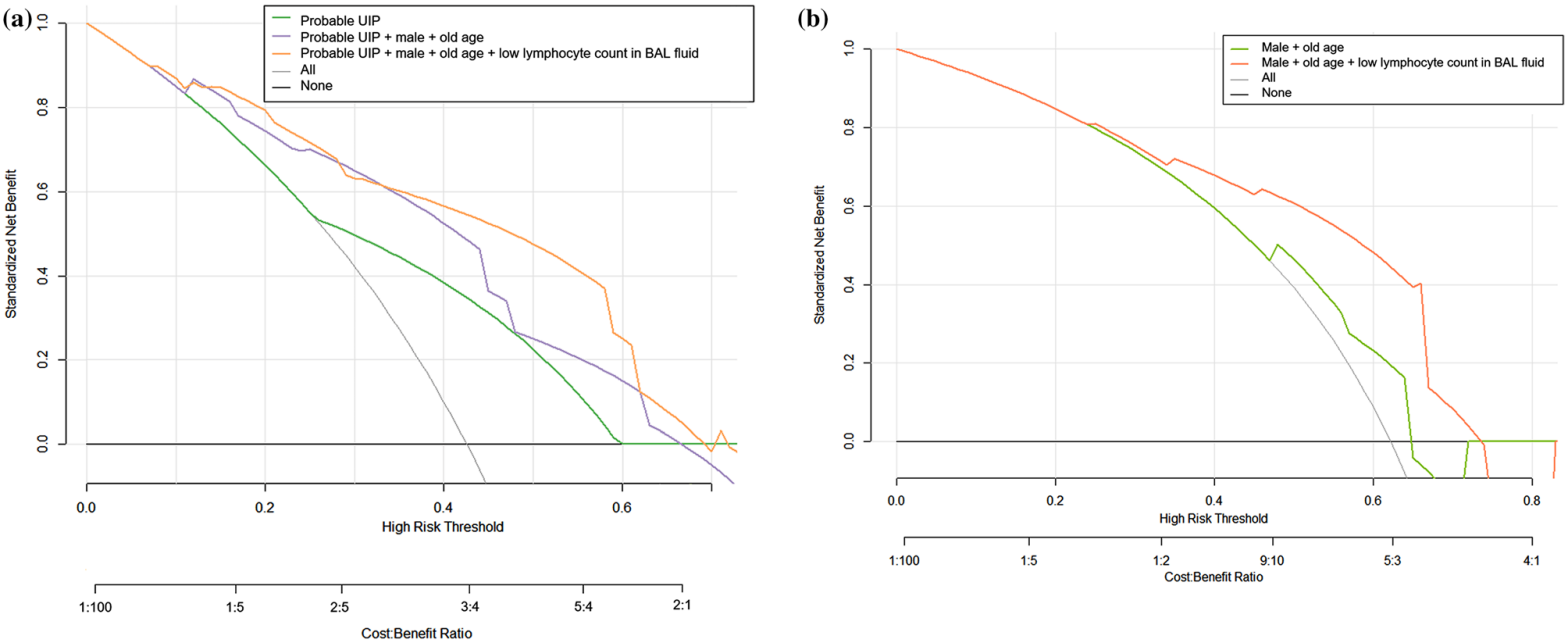
Decision curve analysis of prediction models for the diagnosis of idiopathic pulmonary fibrosis in patients with non-definite CT pattern in the validation cohort. (**a**) Net benefits (proportion of true-positive results minus weighted proportion of false-positive results with weight equal to the ratio of risk threshold to 1 minus risk threshold) of combined model of probable UIP CT pattern, demographic characteristics (sex and age) plus low lymphocyte count in bronchoalveolar lavage fluid were comparable to or higher than those of models with CT pattern alone or combined with age and sex for risk thresholds > 15% in patients with CT pattern of non-definite UIP in the validation cohort. (**b**) Net benefits of combined model of demographic characteristics (sex and age) plus low lymphocyte count in bronchoalveolar lavage fluid were higher than those of models with demographic characteristics alone for risk thresholds > 20% in patients with CT pattern of probable UIP in the validation cohort.

In the validation cohort, the addition of lymphocyte count in BAL fluid to CT pattern alone or together with either older age or male sex improved specificity, PPV and diagnostic accuracy (Table 5). Diagnosing IPF when CT pattern showed probable UIP with lymphocyte count ≤ 15% in BAL fluid, and either male sex or age ≥ 60 years showed a high specificity of 90.6% (95% CI: 79.3–96.9) and a PPV of 80.8% (95% CI: 63.4–91.1). The model incorporating result of BAL fluid analysis had higher net benefits persistently than the other models for risk thresholds > 15% in the validation cohort of non-definite UIP CT pattern and for risk thresholds > 20% in the validation cohort of probable UIP CT pattern (Fig. 3). The net reclassification improvement of the model incorporating result of BAL fluid analysis over the model of age and sex was 0.75 (95% CI: 0.19–1.31) in the validation cohort of probable UIP CT pattern, which showed significant improvement in classification accuracy for IPF diagnosis (*p* = 0.007).

**Table 5:** Test characteristics of probable UIP pattern on CT alone and together with clinical features for IPF diagnosis in patients with CT pattern of non-definite UIP.

	Development cohort (n = 142)						Validation cohort (n = 94)				
Model criteria		Sens	Spec	PPV	NPV	Accuarcy	Sens	Spec	PPV	NPV	Accuarcy
Probable UIP		72.1 (59.2–82.9)	63.0 (51.5–73.4)	59.5 (51.5–67.0)	75.0 (66.0–82.3)	66.9 (58.5–74.6)	68.3 (51.9–81.9)	67.9 (53.7–80.1)	62.2 (51.4–72.0)	73.5 (63.0–81.8)	68.1 (57.7–77.3)
Probable UIP and male with age ≥ 60 years		41.0 (28.6–54.3)	92.6 (84.6–97.2)	80.6 (64.6–90.5)	67.6 (62.6–72.2)	70.4 (62.2–77.8)	24.4 (12.4–40.3)	88.7 (77.0–95.7)	62.5 (39.8–80.8)	60.3 (55.4–64.9)	60.6 (50.0–70.6)
Probable UIP and low lymphocyte count (≤ 15%) in BAL fluid		54.1 (40.9–66.9)	88.9 (80.0–94.8)	78.6 (65.5–87.6)	72.0 (66.0–77.3)	73.9 (65.9–80.9)	55.7 (37.4–69.3)	86.8 (74.7–94.5)	75.9 (59.8–86.9)	70.8 (63.1–77.4)	72.3 (62.2–81.1)
Probable UIP, male with age ≥ 60 years and low lymphocyte count (≤ 15%) in BAL fluid		34.4 (22.7–47.7)	97.5 (91.4–99.7)	91.3 (71.9–97.7)	66.4 (62.1–70.4)	70.4 (62.2–77.8)	17.1 (7.2–32.1)	92.5 (81.8–97.9)	63.6 (35.5–84.8)	59.0 (55.1–62.8)	59.6 (49.0–62.8)
Probable UIP and either low lymphocyte count (≤ 15%) in BAL fluid or male with age ≥ 60 years		60.7 (47.3–72.9)	84.0 (74.1–91.2)	74.0 (62.4–83.0)	73.9 (67.2–79.7)	73.9 (65.9–80.9)	73.3 (60.3–83.9)	73.5 (55.6–87.1)	83.0 (73.2–89.7)	61.0 (49.5–71.3)	73.4 (63.3–82.0)
Probable UIP, low lymphocyte count (≤ 15%) in BAL fluid and either male or age ≥ 60 years		52.5 (39.3–65.4)	90.1 (81.5–95.6)	80.0 (66.5–89.0)	71.6 (65.7–76.8)	73.9 (65.9–80.9)	51.2 (35.1–67.1)	90.6 (79.3–96.9)	80.8 (63.4–91.1)	70.6 (63.4–76.9)	73.4 (63.3–82.0)

Data in parentheses are 95% confidence intervals. HRCT high-resolution computed tomography; *IPF* idiopathic pulmonary fibrosis; *PPV* positive predictive value; *NPV* negative predictive value; *AUC* area under the receiver-operator characteristics curve; *UIP* usual interstitial pneumonia; *BAL* bronchoalveolar lavage.

## Discussion

The present study showed that application of the latest 2018 diagnostic criteria for IPF proposed by ARS/ERS/JRS/ALAT resulted in the reclassification of patients categorized as having possible UIP based on 2011 criteria into two categories, probable UIP and indeterminate for UIP. Prognoses differed significantly among patients grouped by both 2018 and 2011 criteria, with patients classified as indeterminate for UIP according to 2018 criteria showing significantly better survival than the other groups. Although diagnosing IPF based on a CT pattern of probable UIP on 2018 criteria showed increase in sensitivity, its specificity and PPV remained insufficient. The latest ARS/ERS/JRS/ALAT guideline recommend cellular analysis of BAL fluid for patients with newly detected ILD of unknown cause who are clinically suspected of having IPF and have an HRCT pattern of probable UIP, indeterminate for UIP or an alternative diagnosis. In our study, the inclusion of BAL fluid analysis increased diagnostic performance, including specificity and PPV, compared with a model that included CT pattern and appropriate clinical features increasing the likelihood of IPF, i.e. old age and male.

A CT pattern of probable UIP has been found to indicate a higher likelihood of UIP on biopsy^8–10^, especially when compared with a CT pattern of indeterminate for UIP (82.4% vs 54.2%; *p* = 0.01)^11^. Our study found, however, that the proportion of patients diagnosed with IPF did not differ significantly in patients with CT patterns of probable UIP from those with indeterminate for UIP (62.9% vs 78.6%; *p* = 0.05). The discrepant results might be due to different methods used for accounting for the outcome (i.e. histologic UIP vs multidisciplinary diagnosis of IPF) and different proportions of diseases in the tested cohort as there were small numbers of patients with NSIP (9%) and patients with connective tissue disease were not clearly excluded in the Chung et al.’s study^11^. In addition, coexistence of more than moderate degree of emphysema was present in patients who were classified as indeterminate for UIP and the emphysema could affect the evaluation of CT pattern, which could lead to interpret as indeterminate for UIP, finally increasing the proportion of IPF in patients with CT pattern indeterminate for UIP. However, this was not addressed in the 2018 guideline.

This result also affected the diagnostic performance and CT pattern of probable UIP demonstrated a slightly higher specificity (63.3% vs 57.6%) but a lower PPV compared to possible UIP category based on 2011 criteria (62.9% vs 65.8%). As demonstrated in our study, the PPV can be variable and may not be satisfactory regarding the study population of cohort, and among the patients with probable UIP CT pattern, there still can be a significant heterogeneity of underlying disease that certain proportion of iNSIP also can show similar CT findings. This argues the assertion to forgo surgical lung biopsy in patients with probable UIP CT pattern. When we encounter fibrosing ILD without an identifiable cause and suspected to be IPF in clinical practice, the most problematic differential diagnosis, which should be differentiated from IPF would be iNSIP or cHP. Moreover, interobserver agreement for probable UIP was fair (κ = 0.40), which was quite unsatisfying to use as a final confirmative diagnostic criteria in clinical decision making to guide further invasive diagnostic procedures. Among patients with CT pattern of probable UIP, compared to non-IPF patients (i.e. iNSIP), prognosis was also different and the survival time was shorter in IPF patients which was consistent with a previous study^12^. This emphasizes that the patients with CT pattern of probable UIP are still a heterogeneous group, and increasing the clinical likelihood of IPF is important. Diagnosing IPF based on a probable UIP CT pattern should be applied carefully in a selected population with a high clinical likelihood of IPF. Although our study cohort did not include all diseases that can cause fibrosing ILD but only major diseases, we think that our study cohort better reflects the problems regarding the diagnosis of IPF in real clinical practice.

The discriminatory value of BAL in the real-world population is poorly defined and remains controversial even among experts^13–15^. In this study of Asian cohort, adding BAL to rule out the possibility of non-IPF, provide better diagnostic performance for diagnosing IPF in patients with non-definitive UIP. We found that the optimum lymphocyte count cut-off was 15%, a percentage lower than expected. BAL lymphocyte content is similar in IPF and healthy control subjects^16^, and when increased in IPF is associated with moderate to severe alveolar septal inflammation, raising the possibility of an alternative disease, such as cHP, iNSIP or connetive tissue disease-associated ILD. Many studies have demonstrated differences in BAL cell counts between lung diseases , but their variability has always been found to be great^17, 18^, resulting in considerable overlap that might not allow a reliable diagnosis in individual patients regardless of statistical significance. However, it can be used as a safe guard and important complementary method for optimal selection of patients to undergo a diagnostic surgical biopsy in patients with probable UIP CT pattern.

Patients categorized as indeterminate for UIP were a heterogeneous group but including the majority of patients with heavy smokers and patients with emphysema which was in accord with Tzilas et al.^19^ or early stage ILD showing better pulmonary function. Morevoer, regardless of final diagnoses of IPF or non-IPF, patients with CT pattern of indeterminate for UIP had a good prognosis. Our study showed that prognosis of patients with CT pattern of possible UIP can be better stratified based on 2018 criteria.

There are several limations in our study. A main limitation of our study lies in the lack of an external validation cohort which to confirm our findings. However, the scarcity of well-characterised populations of IPF patients, even in tertiary centers, is well recognised. External validation with a cohort of a different prevalence of disease or a prospective study would be helpful to further provide the evidence of routine use of BAL. Second, although we included patients who underwent surgical lung bipsy to build a cohort with less diagnostic uncertainty, it can cause selection bias, mainly in patients with IPF this could include patients with more atypical features compared to patients who did not undergo lung biopsy, which can cause a lower PPV in patients with probable UIP CT pattern. However, the rate of biopsies has changed over time and many patients with typical clinical and imaging features also underwent biopsies in the early period and are included in the study cohort. Third, we did not involve all fibrosing ILD which we can encounter in the real clinical practice and our study cohort may not fully reflect the real prevalence of fibrosing ILD. However, we included iNSIP and cHP since those are the major fibrosing ILD which should be differentiated from IPF in clinical practice. Lastly, as a single center retrospective study, all three readers, thoracic radiologists, were from the same institution, which can limit the generalizability of the results of the study.

In conclusion, the 2018 ATS/ERS/JRS/ALAT diagnostic criteria for IPF provide a better prognostic stratification in patients with CT pattern of possible UIP than 2011 criteria. However, diagnosis of IPF based on CT pattern for probable UIP remains insufficient. Adding BAL fluid results can improve the diagnostic certainty for IPF diagnosis in patients with probable UIP CT patterns.

## Materials and methods

### Study population

This retrospective study was approved by the Institutional Review Board of Asan Medical Center (IRB number 2018-1284), which waived the requirement for written informed consent. Patients newly diagnosed with IPF and a clinically relevant control group consisting of patients with iNSIP and cHP who underwent surgical lung biopsy at Asan Medical Center, Seoul, South Korea, between July 1995 and January 2016 were included (Fig. 1). The final diagnoses were revalidated through multidisciplinary discussion by clinician, radiologists and pathologists with reference to the 2011 ATS/ERS/JRS/ALAT guideline^20^. Clinical data (presentation, antigen exposures, smoking status, associated disease and lung function changes), radiological and histopathological findings were discussed for the final diagnosis. All diagnoses of iNSIP were only made when the histopathologic features suggest fibrotic NSIP with compatible radiological findings^21, 22^. A diagnosis of cHP was only made when the histopathological features were typical showing the presence of poorly formed nonnecrotizing granulomas with chronic fibrosing interstitial pneumonia or airway-centered fibrosis on surgical lung biopsy^23, 24^. For diagnosis of cHP, the presence of an inciting antigen, compatible HRCT imaging, and typical histopathologic findings were considered during the multidisciplinary discussion. The results of BAL fluid analysis were not considered for the multidisciplinary diagnosis in our institution. In our center, the clinical diagnosis of IPF is reached by first excluding other known causes of ILD, and then by the presence of UIP pattern on HRCT in patients not subjected to surgical lung biopsy. In patients who underwent surgical lung biopsy, the diagnoses are based on combinations of HRCT and the surgical lung biopsy pattern followed by multidisciplinary discussion. The approximate rate of biopsy in patients with IPF in our center was about 30% between 2004 and 2017 as reported in a previously published study^25^. However, the rate of biopsies has changed over the long study inclusion period from 1995 to 2016, many patients with typical clinical and imaging features also underwent biopsies in the early period of the study inclusion. In IPF patients of our study cohort, histopathological UIP pattern was present on surgical lung biopsy in patients with CT pattern of alternative diagnosis, and either the histopathological UIP pattern or probable UIP pattern was present in patients with CT pattern indeterminate for UIP. Patients with auto-immune features (i.e., marked lymphoid follicles or plasmacytosis) were excluded priorly and were not screened for study inclusion as we only included a confirmed diagnosis of IPF, iNSIP and cHP. However, 4 patients were excluded who were finally diagnosed with a connective tissue disease and 6 patients were excluded as unclassifiable ILD during the course of a retrospective revalidation of their diagnosis. Patients who were unavailable for histopathologic evaluation or baseline CT and those who were proven to have an isolated cellular NSIP were excluded. Clinical data, including age, sex, smoking history, the results of 6 min walk tests, pulmonary function tests, BAL fluid analysis, histopathologic findings of surgical lung biopsy, and survival were collected. BAL was was performed in accordance with the guidelines^26^. All methods were performed in accordance with the relevant guidelines and regulations.

### CT evaluation

HRCT scans were perfomed in both supine and prone positions. HRCT were indepedently evaluated by three thoracic radiologists (K.D., E.J.C. and J.C., with 18, 15 and 4 years of experience in thoracic radiology, respectively), who were blinded to clinical data and final diagnosis. HRCT scans were simultaneously classified into three categories (UIP, possible UIP or inconsistent with UIP) according to 2011 criteria, or into four categories (UIP, probable UIP, indeterminate for UIP or alternative diagnosis) according to 2018 criteria^5, 20^. For the discordant cases of all three readers, we performed additional reading session and discordant results resolved by consensus. The extent of emphysema was evaluated as mild (1–10%), moderate (11–25%), severe (26–50%) and very severe (> 50%).

### Statistical analysis

All statistical analyses were performed using SPSS (IBM SPSS Statistics for Windows, Version 21.0, IBM Corp., Armonk, N.Y., USA, www.ibm.com/products/spss-statistics) and R (R: A language and environment for statistical computing, R Foundation for Statistical Computing, Vienna, Austria, www.R-project.org) software. To assess the differences in variables between groups, the t-tests or Mann–Whitney U-tests was used for continuous variables and the χ2 test was used for categorical variables. The generalized interobserver agreement for all three readers was evaluated using Fleiss’ κ. Cox proportional hazards models with adjusted survival curves were used to evaluate the associations between CT pattern and survival time. The prognostic impacts were adjusted by age, gender, treatment history of anti-fibrotic agent, baseline FVC and baseline DL_CO_.

To evaluate the added value of BAL fluid analysis for diagnosing IPF in patients with non-definite UIP CT patterns (probable UIP, indeterminate for UIP and alternative diagnosis), such patients were randomly divided into development and independent validation cohorts (3:2 ratio). The correlations with a IPF diagnosis and the results of BAL fluid analysis, along with CT pattern and clinical characteristics such as old age (≥ 60 years) and male sex, were assessed by logistic regression analyses^5^. The best-cutoff for cell count (%) in BAL fluid in the development cohort was determined using Youden’s index^27^. The sensitivity, specificity, PPV, and NPV for the diagnosis of IPF were calculated for each regression model. Diagnostic performance was quantified by measuring the AUC, and to quantify the improvement of usefulness added by BAL fluid analysis, a net reclassification improvement was also evaluated^28^. The clinical utility of each prediction model was assessed by decision curve analysis, by quantifying the net benefits at different threshold probabilities^29, 30^. A *p* value < 0.05 was considered statistically significant.

## Supplementary Information

Supplementary Information.
