# Supplementary material for: Diagnostic and prognostic implications of 2018 guideline for the diagnosis of idiopathic pulmonary fibrosis in clinical practice
Source: Sci Rep. 2021 Aug 13;11:16481. doi: 10.1038/s41598-021-95728-7 (PMC8363716; doi:10.1038/s41598-021-95728-7)

**Diagnostic and prognostic implications of 2018 guideline for the diagnosis of idiopathic pulmonary fibrosis in clinical practice**

Original Research

Jooae Choe^1†^, Byoung Soo Kwon^2†^, Kyung-Hyun Do^1^, Hee Sang Hwang^3^, Jin Woo Song^4*^, Eun Jin Chae^1*^

^1^Department of Radiology, University of Ulsan College of Medicine, Asan Medical Center, Seoul, South Korea.

^2^Division of Pulmonary and Critical Care Medicine, Department of Internal Medicine, Seoul National University Bundang Hospital, Seongnam-Si, Gyeonggi-Do, South Korea.

^3^Department of Pathology, University of Ulsan College of Medicine, Asan Medical Center, Seoul, South Korea.

^4^Department of Pulmonology and Critical Care Medicine, University of Ulsan College of Medicine, Asan Medical Center, Seoul, South Korea.

^†^Jooae Choe and Byoung Soo Kwon contributed equally to this study as co-first authors.

*Eun Jin Chae and Jin Woo Song contributed equally to this study as co-corresponding authors.

**Corresponding Author**

**Eun Jin Chae, MD, PhD**Department of Radiology and Research Institute of Radiology, University of Ulsan College of
Medicine, Asan Medical Center, 88 Olympic-ro 43 Gil, Songpa-gu, Seoul 138-736, Korea.
E-mail: [ejinchae@gmail.com](mailto:ejinchae@gmail.com)

**Jin Woo Song, MD, PhD**Department of Pulmonology and Critical Care Medicine, University of Ulsan College of Medicine, Asan Medical Center, 88 Olympic-ro 43 Gil, Songpa-gu, Seoul 138-736, Korea.
E-mail: jwsongasan@gmail.com

**Supplementary materials**

**Supplementary table 1. CT patterns according to 2011 and 2018 criteria segregated by multidisciplinary diagnoses (MDD)**

| CT pattern | | 2011 criteria | | | 2018 criteria | | | |
| --- | --- | --- | --- | --- | --- | --- | --- | --- |
|  |  | UIP | Possible UIP | Inconsistent with UIP | UIP | Probable UIP | Indeterminate for UIP | Alternative diagnosis |
| MDD | IPF | 165 | 144 | 30 | 165 | 110 | 33 | 31 |
|  | iNSIP | 9 | 51 | 38 | 9 | 48 | 2 | 39 |
|  | cHP | 10 | 24 | 64 | 10 | 17 | 7 | 64 |

CT, computed tomography; MDD, multidisciplinary diagnosis; UIP, usual interstitial pneumonia; iNSIP, idiopathic nonspecific interstitial pneumonia; cHP, chronic hypersensitivity pneumonitis.

**Supplementary table 2. Demographic and clinical characteristics of patients in the development and validation cohorts (subgroup analysis of patients with non-definitive CT patterns who underwent bronchoalveolar lavage fluid analysis)**

| Characteristics | Development cohort  (n = 142) | Validation cohort  (n = 94) | *p-*value |
| --- | --- | --- | --- |
| Age, years | 60.5 ± 9.2 | 61.3 ± 9.0 | 0.487 |
| Male sex | 77 (54.2) | 50 (53.2) | 0.876 |
| Ever smoker | 69 (48.6) | 45 (47.9) | 0.914 |
| Smoking pack-year, years | 15.3 ± 18.2 | 20.1 ± 25.9 | 0.385 |
| 6 min walking distance, m | 447.3 ± 89.6 | 446.0 ± 104.5 | 0.932 |
| DL_CO_% predicted | 61.0 ± 16.0 | 63.0 ± 16.7 | 0.216 |
| FEV_1_% predicted | 77.5 ± 15.1 | 77.6 ± 16.9 | 0.718 |
| FVC% predicted | 72.0 ± 15.3 | 70.6 ± 16.3 | 0.536 |
| Bronchoalveolar lavage, median (IQR)^†^ |  |  |  |
| Lymphocytes (%) | 16 (7-31) | 12 (6-22) | 0.120 |
| Neutrophils (%) | 3 (1-8) | 5 (2-10) | 0.161 |
| Eosinophils (%) | 2 (0-5) | 2 (0-4) | 0.929 |
| Multidisciplinary diagnosis |  |  | 0.148 |
| IPF | 62 (43.7) | 40 (42.6) |  |
| iNSIP | 35 (24.6) | 22 (23.4) |  |
| cHP | 45 (31.7) | 32 (34.0) |  |

Data presented as mean ± SD or number (%).

UIP, usual interstitial pneumonia; NSIP, idiopathic nonspecific interstitial pneumonia; cHP, chronic hypersensitivity pneumonitis; DL_CO_, diffusing capacity of the lung for carbon monoxide; FEV_1_, forced expiratory volume in 1 s; FVC, forced vital capacity.

**Supplementary table 3. Logistic regression prediction models of idiopathic pulmonary fibrosis in the development cohort (subgroup analysis of patients with non-definitive CT pattern who underwent bronchoalveolar lavage fluid analysis; n = 142)**

| Variables | Odds ratio | 95% CI | *p*-value |
| --- | --- | --- | --- |
| Univariable logistic regression |  |  |  |
| CT pattern, probable UIP | 4.40 | 2.14-9.03 | 0.001 |
| Age, ≥60 years | 3.28 | 1.56-6.86 | 0.002 |
| Male (Female) | 4.95 | 2.37-10.32 | < 0.001 |
| BAL low lymphocyte count, ≤15% | 4.18 | 2.06-8.51 | 0.001 |
| Multivariable logistic regression |  |  |  |
| CT pattern, probable UIP | 3.58 | 1.55-8.26 | 0.003 |
| Male (Female) | 5.28 | 2.24-12.45 | 0.001 |
| Age, ≥60 years | 3.53 | 4.44-8.68 | 0.006 |
| BAL low lymphocyte count, ≤15% | 3.58 | 1.57-8.20 | 0.003 |

Data in parentheses are reference values.

UIP, usual interstitial pneumonia; BAL, bronchoalveolar lavage.

**Supplementary table 4. Logistic regression prediction models of idiopathic pulmonary fibrosis in the development cohort (subgroup analysis of patients with probable UIP CT pattern who underwent bronchoalveolar lavage fluid analysis; n = 74)**

| Variables | Odds ratio | 95% CI | *p*-value |
| --- | --- | --- | --- |
| Univariable logistic regression |  |  |  |
| Age ≥60 years | 2.25 | 0.79−6.39 | 0.127 |
| Male (Female) | 6.00 | 2.16−16.65 | < 0.001 |
| Male with age ≥60 years | 5.26 | 1.80−15.43 | 0.003 |
| BAL low lymphocyte count ≤15% | 7.00 | 2.48−19.75 | < 0.001 |
| Multivariable logistic regression |  |  |  |
| Male (Female) | 4.07 | 1.36−12.23 | 0.012 |
| BAL low lymphocyte count, ≤15% | 4.98 | 1.66−14.93 | 0.004 |

Data in parentheses are reference values.

UIP, usual interstitial pneumonia; BAL, bronchoalveolar lavage.

**Supplementary figure legends**

**Supplementary figure 1. Kaplan–Meier curves of overall survival stratified according to the CT pattern of 2011 and 2018 guidelines for diagnosis of idiopathic pulmonary fibrosis.**

a, b) Total patients with fibrosing interstitial lung disease and c, d) patients with idiopathic pulmonary

fibrosis as a function of CT pattern according to 2011 and 2018 diagnostic criteria, respectively.

1. (b)


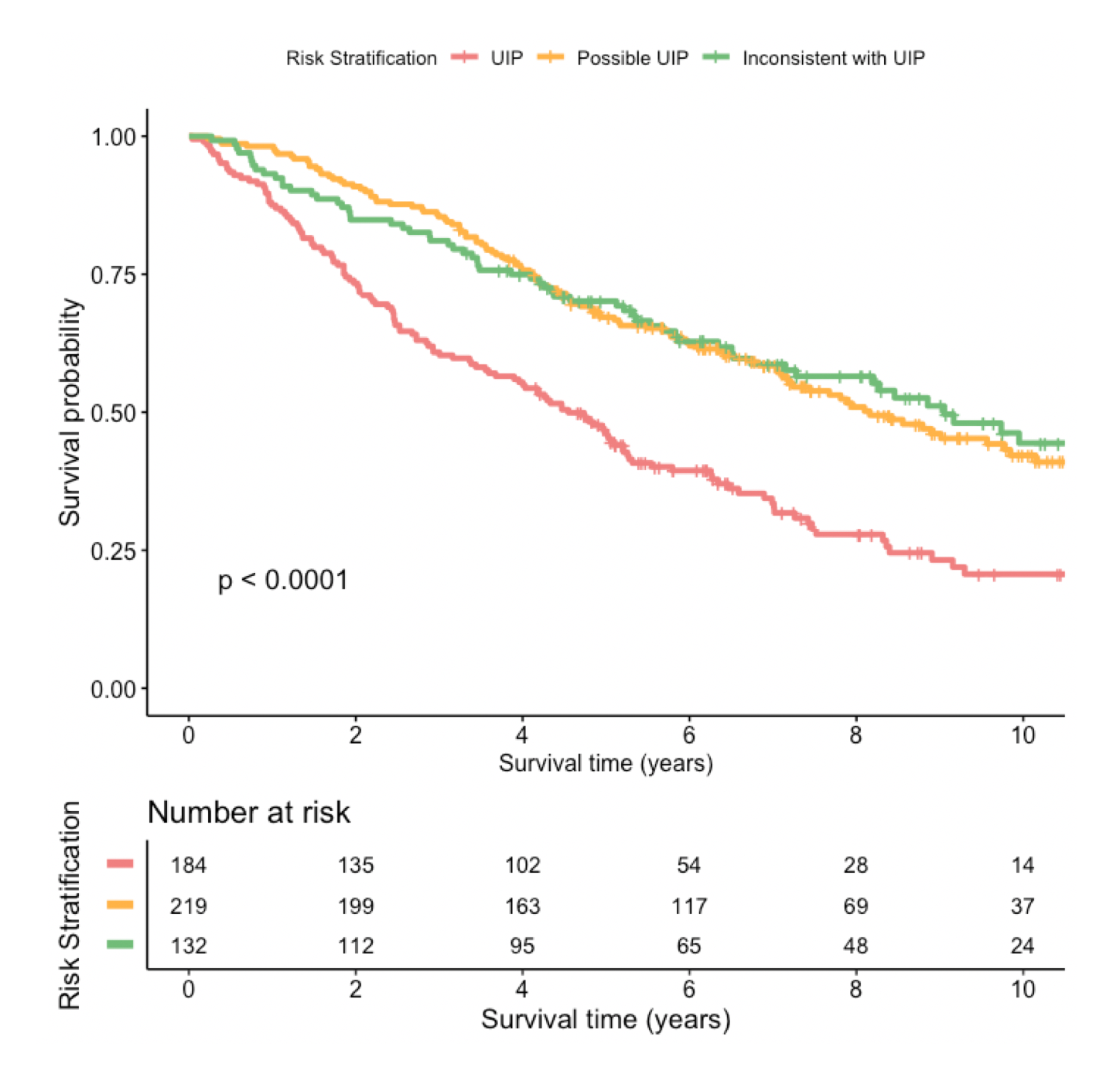

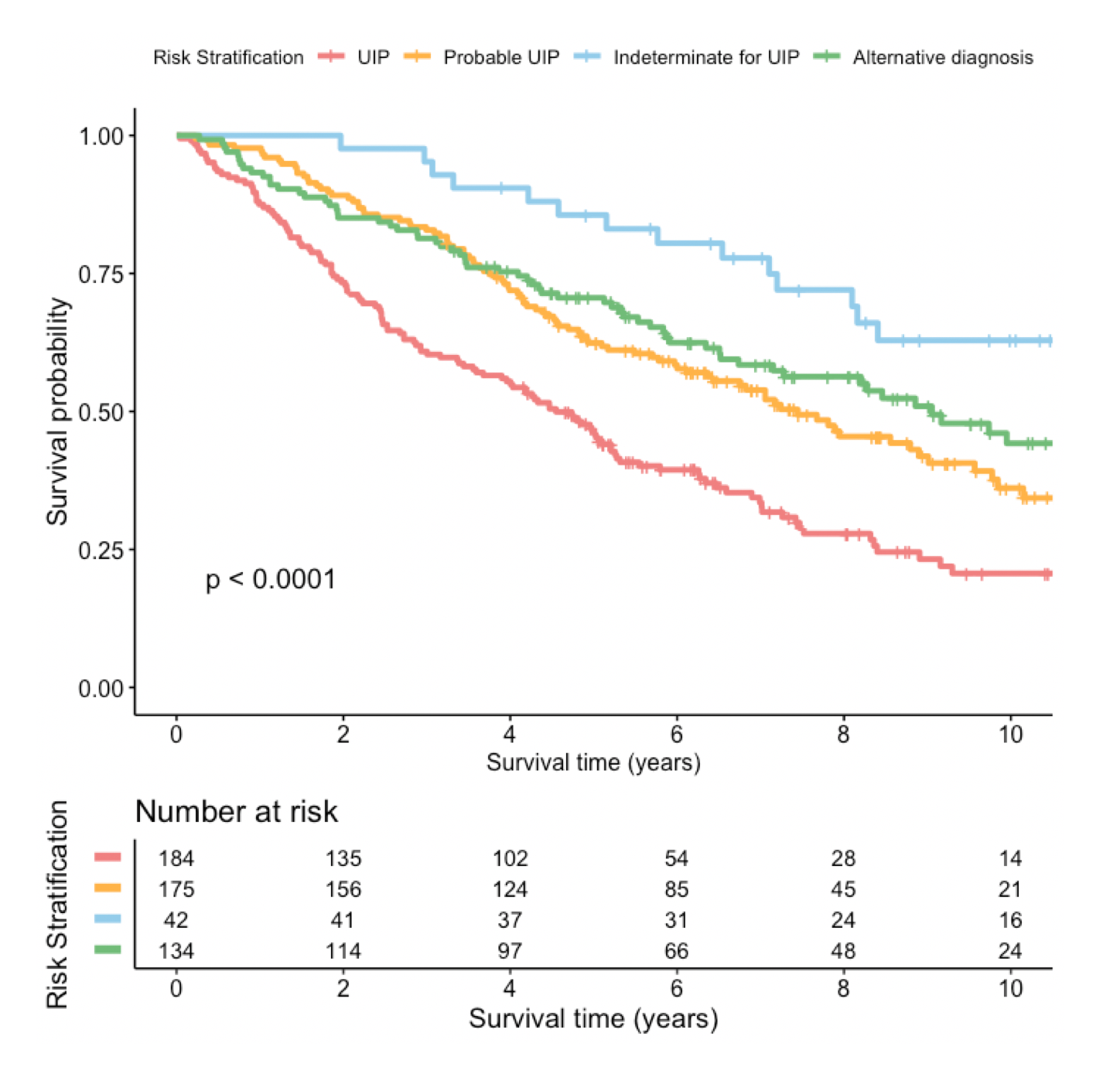


1. (d)


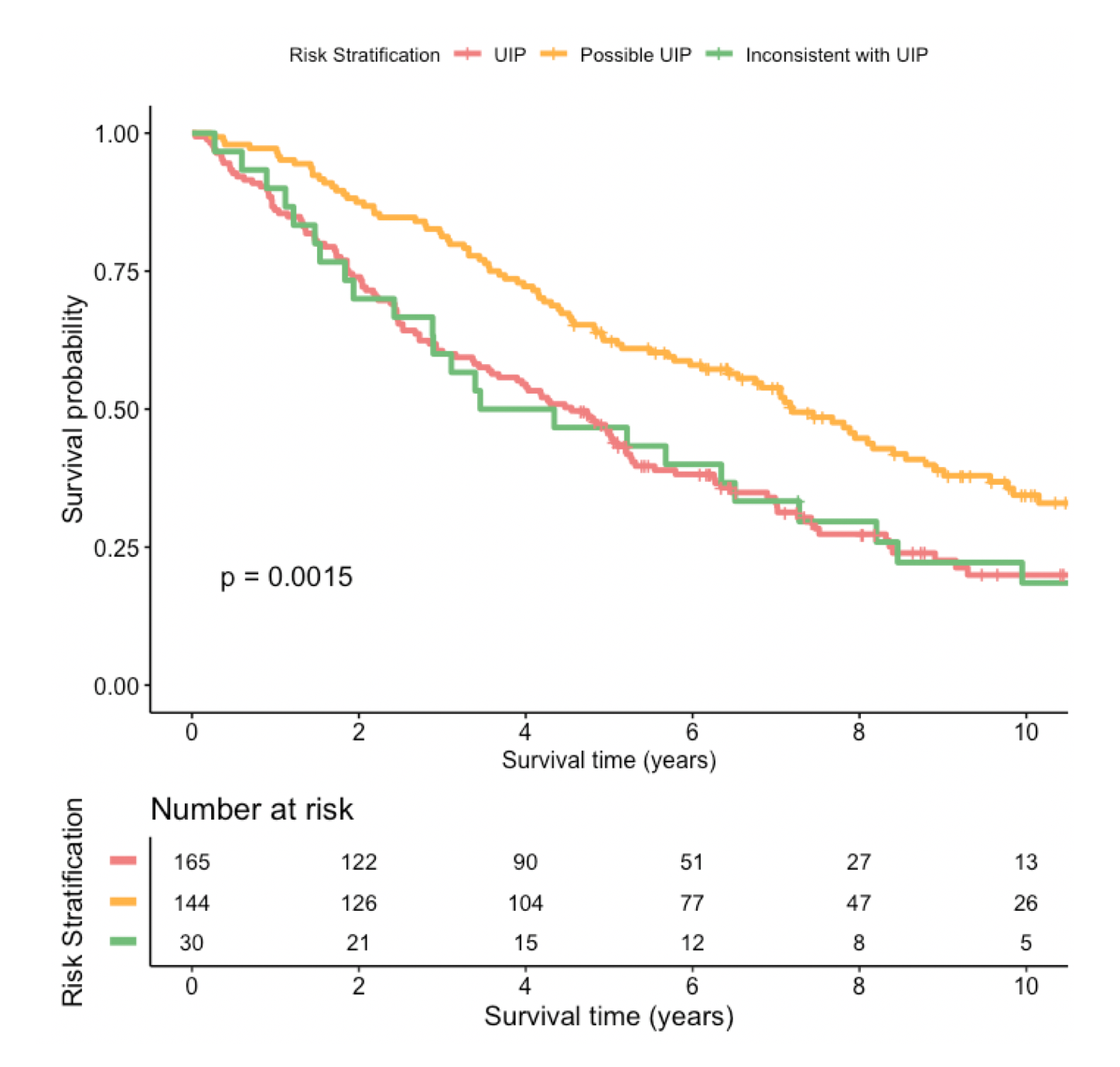

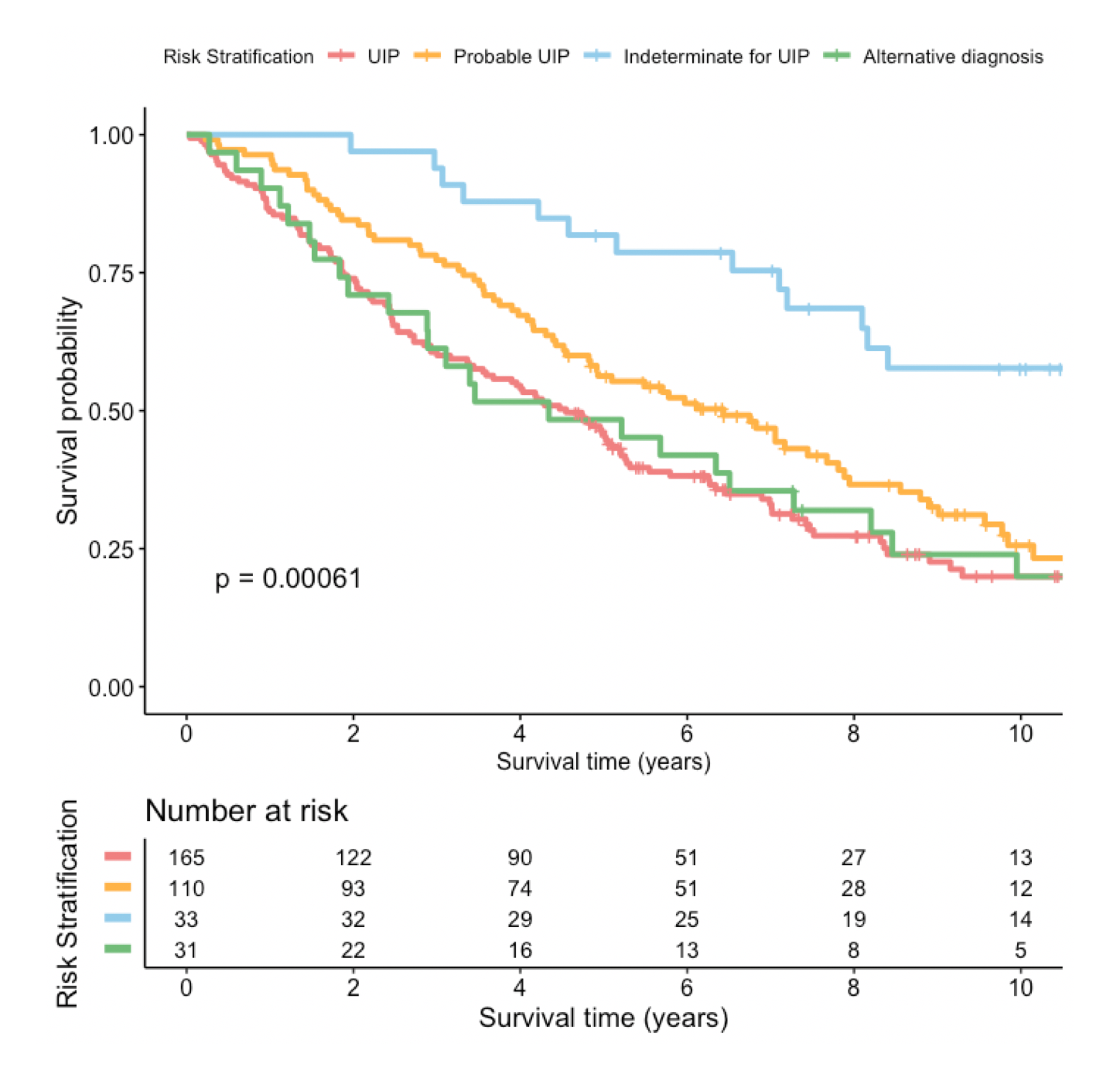


**Supplementary figure 2. Box-plots of lymphocyte and neutrophil count (%) in bronchoalveolar lavage (BAL) fluid.**

The percentage of lymphocytes was significantly lower in the idiopathic pulmonary fibrosis (IPF) group compared to non-IPF group (p<0.001) in both a) development and b) validation cohort. There was no significant difference in the percentage of neutrophils between the IPF and non-IPF groups in both c) development and d) validation cohorts.

1. (b)


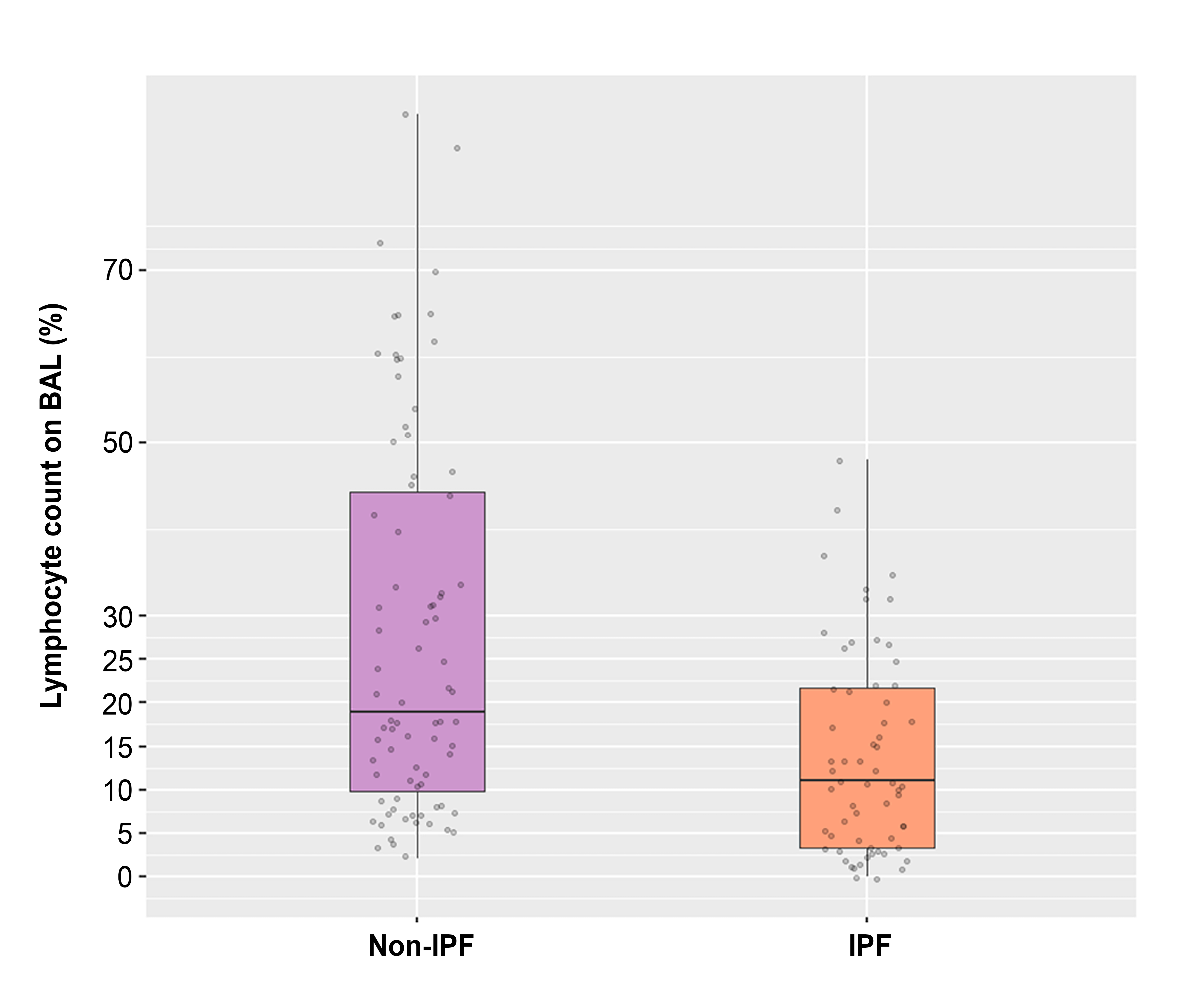

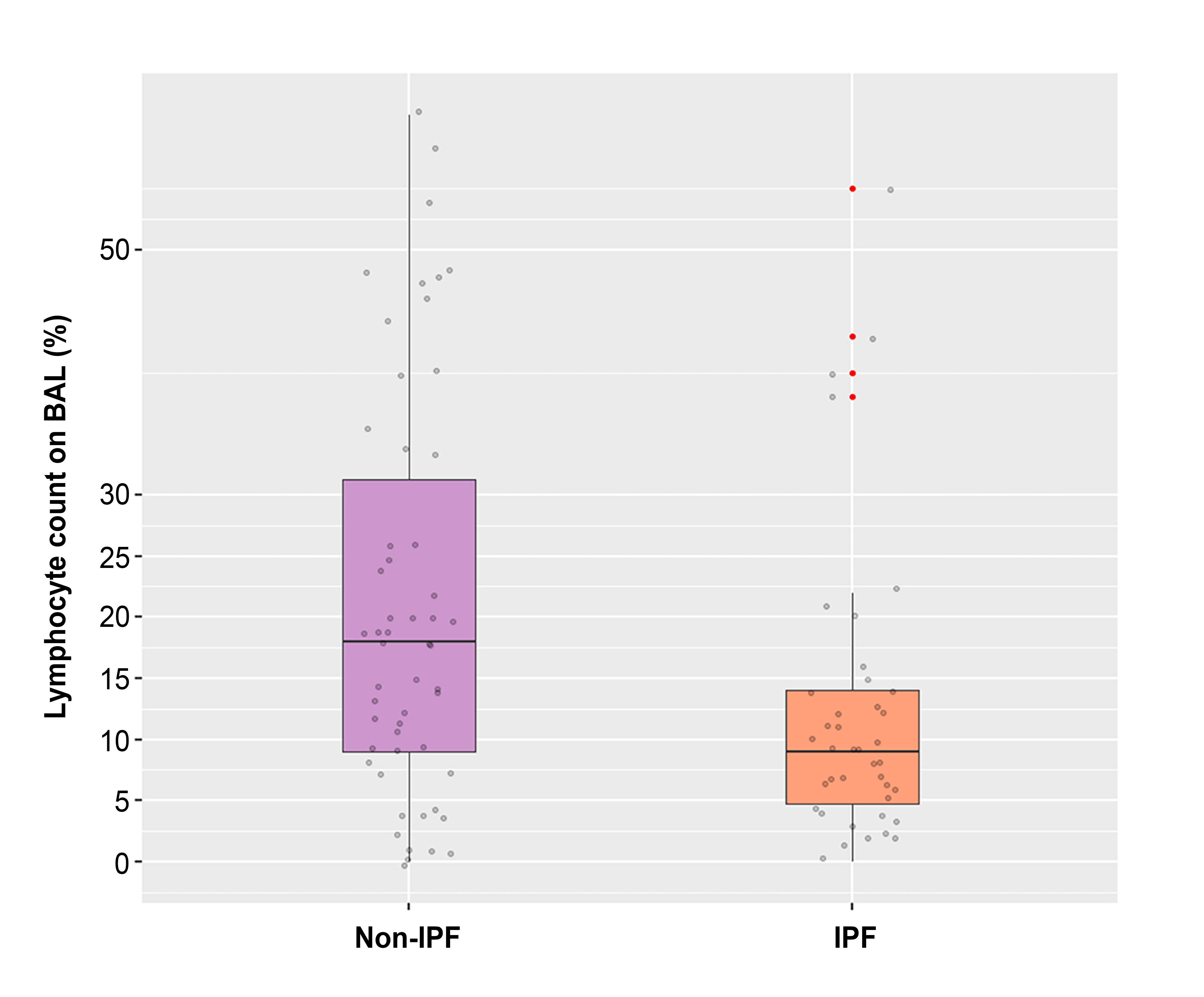


1. (d)


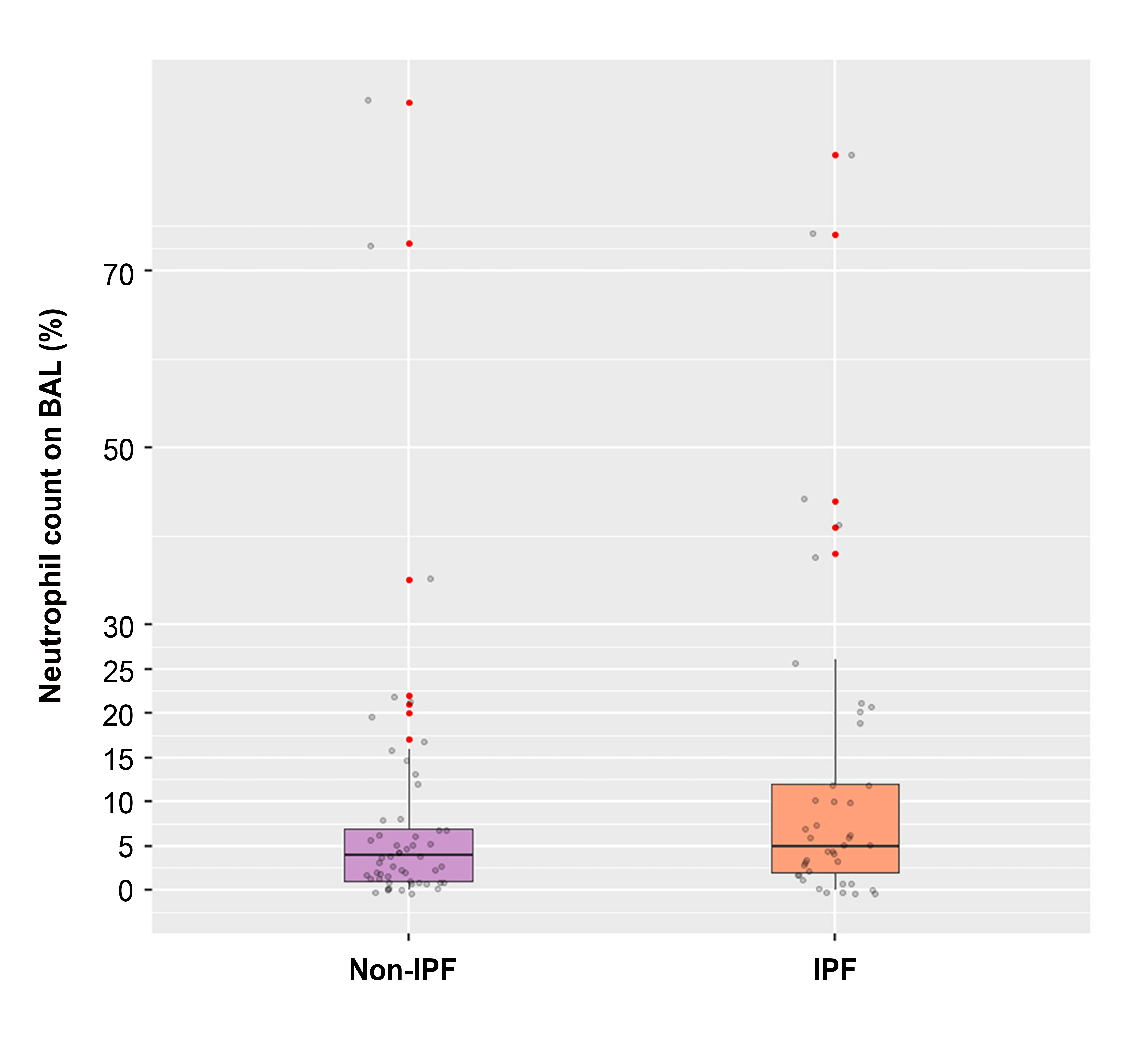

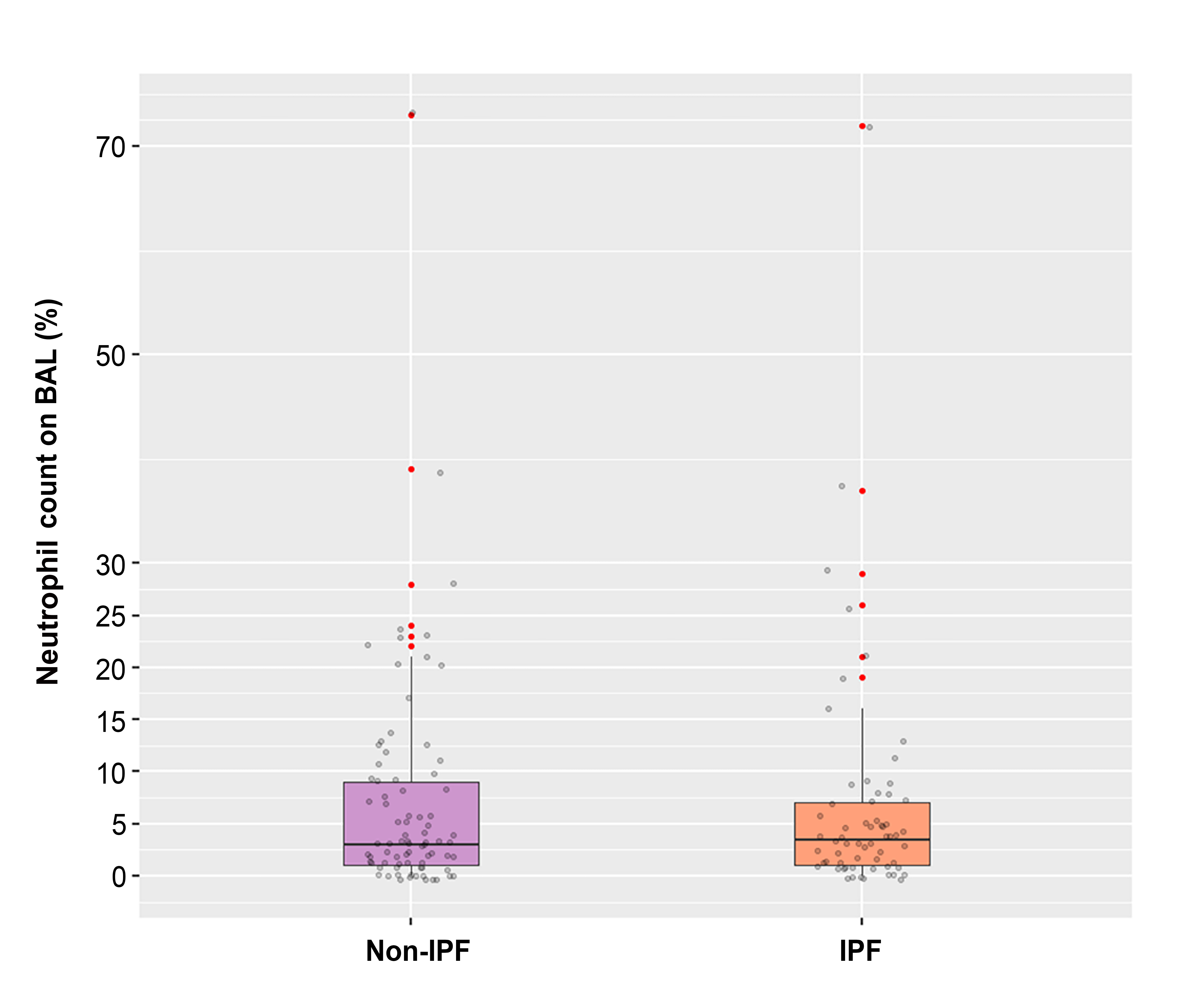

Supplement: Supplementary file 1 — Supplementary Information. [file 41598_2021_95728_MOESM1_ESM.docx]
